# Microplastics exposure altered hematological and lipid profiles as well as liver and kidney function parameters in albino rats (*Rattus norvegicus*)

**DOI:** 10.5620/eaht.2024021

**Published:** 2024-06-26

**Authors:** Tajudeen Olanrewaju Yahaya, Abdulgafar Bala Ibrahim, Abdulrahman Sani Kalgo, Mutiyat Kehinde Adewale, Chikelu Chinelo Emmanuela, Baliqees Abdulkadir, Adamu Zainab Fari, Asiya Koko Attahiru, Abdullahi Saadatu, Joseph Dahali Wanda

**Affiliations:** 1 Department of Biological Sciences, Federal University Birnin Kebbi, Kebbi State, Nigeria

**Keywords:** Hematological parameters, Kidney function parameters, Lipid profiles, Liver function parameters, Microplastics

## Abstract

The global occurrence of microplastics and their poorly understood health implications underscore the need for scientific investigation. This study aimed to assess the effects of microplastics exposure. Twenty-five (25) albino rats (*Rattus norvegicus*) were divided into five (5) groups, each consisting of five rats. Group 1 (the negative control) received normal feed; group 2 (the positive control) was administered a 10 % lead acetate solution; and groups 3, 4, and 5 were administered 1 %, 5 %, and 10 % microplastic solutions, respectively. The rats were monitored for 28 days, after which blood samples were taken for hematological and lipid profiles as well as liver and kidney function parameters. The results revealed dose-dependent significant (p < 0.05) alterations in the health indices of the treated rats and the positive control compared with the negative control. Specifically, the hematological parameters, including the white blood cells (WBC) and its subtypes, were reduced, indicating immunosuppressive effects, and the red blood cells (RBC), hemoglobin (HGB), hematocrit (HCT), platelets, mean corpuscular volume (MCV), mean corpuscular hemoglobin (MCH), and mean corpuscular hemoglobin concentration (MCHC) were reduced, indicating anemia. The 1 % and 5 % microplastic solutions raised the lipid profiles of the treated rats, including total cholesterol (TC), triglycerides (TG), high-density lipoprotein (HDL), and low-density lipoprotein (LDL), while the 10 % concentration decreased them, causing hyperlipidemia and hypolipidemia, respectively. The liver function parameters, including total protein (TP), albumin (ALB), aspartate transaminase (AST), alanine transaminase (ALT), and alkaline phosphatase (ALP), were elevated, indicating liver damage. Elevation of kidney function parameters, including sodium ion (Na^+^), potassium ion (K^+^), chloride ion (Cl^-^), urea, and creatinine (CRT), were noticed, suggesting kidney injuries. It can be inferred from these results that microplastics are toxic. Hence, human exposure to microplastics should be reduced to a minimum.

## Introduction

Microplastics, tiny particles with a size range of <5 µ m, are pervasive globally, spanning from polar regions to the equator, and encompassing coastal areas and aquatic ecosystems [1,2]. Their non-biodegradability and persistent nature contribute to their widespread distribution and accumulation in the environment [3-5]. Additionally, their dispersion is facilitated by transport phenomena such as wind and ocean currents [2]. Moreover, the escalating production of plastics to meet the rising demand for packaging materials is increasing their occurrence. Since the year 2000, annual global plastic production has exceeded 200 million tons, surpassing 300 million tons in 2020, and is projected to exceed 500 million tons in 2030 [2]. The inefficient disposal of these plastics, with global recovery and recycling rates below 10 %, results in an estimated 22 million tons of plastic waste entering the environment annually, with a considerable portion being single-use plastic [6].

While some of these plastics end up in terrestrial environments, the majority find their way into aquatic ecosystems. These plastics undergo photodegradation, transforming into microplastics (classified as secondary microplastics), accumulating in the soil [7], water and sediment [8], and terrestrial and aquatic organisms [9]. Additionally, certain microplastics classified as primary microplastics are intentionally produced for various products like textiles, cosmetics, and fishing nets, entering the environment or human bodies [8]. These microplastics can lodge in the digestive tracts and tissues of many wildlife species, including fish and shellfish [10]. Through the food web, some of these microplastics enter human systems, having been detected in human stools, tissues, brain, breast milk, and organs [11]. Moreover, microplastics can become airborne, lodging in the respiratory tracts of animals, including humans [12]. Consequently, microplastics pose a significant risk to human health [13].

Nigeria, being the most populous African country, stands as a significant consumer of plastics and ranks ninth for plastic pollution, generating approximately 2.5 million tons of plastic waste annually, with less than 12% undergoing recycling [14]. Moreover, over 60 million plastic sachets of water are consumed and disposed of daily in Nigeria [15]. In the country, microplastics have been detected in marine water and sediments [8], freshwater, sediments, and aquatic organisms [16], borehole water [17], and bottle water [18], among others. This indicates that microplastic pollution presents a great risk in the country, and authorities must act promptly. To this end, governments are currently focusing attention on microplastic pollution, and Lagos State, in particular, has banned single-use plastic products. However, due to its ubiquity, microplastic exposure is unavoidable, and the effort being made must be complemented with therapeutic strategies for the exposed. To achieve this, the exact nature of the health consequences of microplastics must be ascertained. Unfortunately, available literature shows that there is a dearth of studies on the health consequences of microplastic exposure in the country.

Globally, existing studies on microplastics primarily focus on the qualitative and quantitative aspects of the pollutants in the environment; thus, little is known about their health risks [19]. Scientists, however, have theorized and categorized potential health risks into physical and chemical effects [20]. Physical effects are influenced by microplastics' sizes, shapes, and concentrations, while chemical effects depend on the toxic compounds in the microplastics, including additives, polymeric raw materials, chemicals absorbed from the environment such as heavy metals, as well as microorganisms and biomolecules such as allergens and antibiotics [20,21,22]. Thus, there is a need for studies focusing on the health effects of microplastics. This will enable health professionals to devise ameliorative and therapeutics measures for microplastic exposure. Such health evaluations can be conducted using frequently employed health indices, particularly hematological parameters and serum biochemistry parameters such as lipid profile, liver, and kidney functions in exposed animals. These parameters serve as sensitive indicators of the physiological, health, and metabolic status of animals, and their changes are crucial in assessing responses to environmental factors [23,24]. The main objective of this study was to evaluate the health consequences of microplastics exposure by assessing the hematological parameters, lipid profiles, and liver and kidney function of albino rats (*Rattus norvegicus*) exposed to microplastic solutions.

## Materials and Methods

### Description of the study area

**Figure 1.**
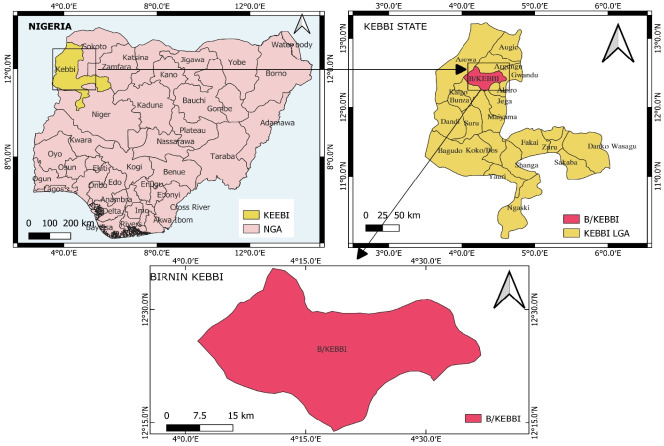
Map of the study area (drawn with ArcGIS version 10.3).

This study was conducted in Birnin Kebbi, the capital of Kebbi State, situated in northwestern Nigeria at the intersection of roads from Argungu, Jega, and Bunza. The city lies at a latitude of 12.466078° N and a longitude of 4.199524° E (Figure 1). Birnin Kebbi also serves as the headquarters of the Gwandu Emirate. The estimated population of Birnin Kebbi in 2024 is 428,676 [25]. Kebbi State shares borders with Niger Republic to the west, Benin Republic to the southwest, and also borders the Nigerian states of Sokoto and Zamfara to the north and east, respectively. The city is predominantly inhabited by indigenous tribes, mainly Hausa, Fulani, and Zuru people, as well as significant populations of settlers, mainly Yoruba, Igbo, and Nupe ethnic tribes. The climate of Birnin Kebbi is characterized by a long dry season from October to May, with the rainy season lasting for five months (June to October). The annual rainfall ranges from 762 to 1016 mm, with the heaviest rainfall in August [26]. The area also experiences harmattan, associated with a cold, dusty wind blowing from November to February [26]. During the harmattan period, temperatures drop to about 18 °C to 20 °C [26]. However, the average daily temperature of the city can reach as high as 45 °C during hot weather. The natural vegetation of the area is sparse, with trees about 10 - 12 m tall widely spaced, typical of Sudan Savannah.

Plastic materials such as water sachets, water bottles, polybags, buckets, packaging, among others, are extensively used in the city, as is common worldwide. However, these materials are indiscriminately dumped in the environment and could be contaminating soil, groundwater, plants, and animals. Humans could also breathe in the particles or ingest them through drinking water or food web. Plastic debris could be impacting human lives in the city, yet globally, the health effects of plastics have not been fully understood, necessitating the current study.

### Animal protocol

Twenty-five (25) albino rats (*Rattus norvegicus*) of both sexes, aged 65 days and weighing 180 ± 3.32 g, were obtained from the animal house of Usmanu Danfodiyo University, Sokoto, Nigeria, in September 2023. Subsequently, the rats were housed in metallic cages and provided with commercial pellets (produced by Vital Feeds, Lagos) and water *ad libitum*, served in stainless steel bowls and plates. Standard laboratory conditions were maintained at a normal room temperature of 26 ˚C under a light-dark cycle of 12:12 hours. The rats underwent a three-week domestication period before the commencement of the experiment.

The guide of care and use of animals in research and teaching of the Animal Ethics Committee of Federal University Birnin Kebbi, Nigeria in line with the National Institutes of Health guide for the care and use of Laboratory animals (NIH Publications No. 8023, revised 1978), were followed in maintaining the rats. Approval for the experimental procedures was obtained with the ethical code FUBK/AEC/FS/M1052.

### Source and preparation of microplastics

Microplastic solutions were prepared following the procedures outlined by Vortmann et al. [27] with some modifications. New polyethylene and polypropylene plastics were procured from a local store in Kalgo, Kebbi State, in September 2023. Subsequently, the plastics underwent a washing process with distilled water to eliminate impurities and were then dried for 48 hours to facilitate crushing and blending. The plastics were thoroughly crushed using a CALIFORNIA Desire 550-watt electric grinder for 15 minutes in each round. The resulting particles were then passed through a 2 mm mesh sieve within the microplastic size range. Furthermore, the powdered material was examined under a microscope and confirmed to be in the size range of 0.1 μm to 2 mm. Fourier-transform infrared spectroscopy was employed to characterize the microplastics, confirming that they contained polyethylene and polypropylene polymers. Their dominant shapes were fragments and pellets. The powder was subsequently stored in glass vials, and various concentrations of the microplastic solution were obtained by weighing with a digital balance (manufactured by KERN, Serial No: W 1400949). The concentrations made are as follows: 1 % (1 g of microplastics in 100 ml of ultrapure water), 5 % (5 g of microplastics in 100 ml of ultrapure water), and 10 % (10 g of microplastics in 100 ml of ultrapure water).

Additionally, a 10 % lead acetate solution was prepared to serve as positive control. Lead acetate is a known hematobiochemical toxicant as demonstrated by Ilesanmi et al. [28].

### Experimental design

The 25 rats were randomly distributed into five groups, each consisting of 5 rats. The negative control rats, labelled as group 1, were provided with normal feed and water, while the positive control rats in group 2 were administered 10% lead acetate. The test rats in groups 3, 4, and 5, respectively, were orally administered 1 %, 5 %, and 10 % microplastic solutions for a duration of 28 days. Thereafter, the rats were subjected to overnight fasting for 12 hours, after which blood samples were collected to assess hematological parameters, lipid profile, as well as liver and kidney function parameters.

### Collection of blood sample

The rats were euthanized by asphyxiation using carbon dioxide followed by cervical dislocation [29]. Exactly 3.5 ml of blood sample was drawn using a 5 ml syringe and a 20-gauge needle into sample bottles. Four blood samples were taken from each group to cover the four tests to be carried out. The sample bottles intended for the estimation of haematological parameters were pre-filled with ethylenediaminetetraacetic acid (EDTA) to prevent coagulation. They were immediately inverted 8-10 times after collection to mix and ensure adequate anticoagulation of the specimen. Sample bottles intended for other tests were not pre-filled with EDTA.

### Haematological test

Blood smear slides were prepared for microscopic examination. Subsequently, the Spincell 3 automated hematology and biochemistry analyzer Urit 3000 Plus, version 07/2015 model, was utilized to count red blood cells (RBC) and white blood cells (WBC), as well as platelets (PLT). Additionally, a differential count was performed to identify various white blood cell subtypes, including lymphocytes (LYM) and neutrophils (NEUT). Hemoglobin (HGB) levels were determined spectrophotometrically, while hematocrit (HCT), the ratio of RBC to the total blood volume; mean corpuscular volume (MCV), the ratio of HCT/RBC; mean corpuscular hemoglobin (MCH), the ratio of HGB/RBC; and mean corpuscular hemoglobin concentrations (MCHC), the ratio of HGB/HCT, were calculated.

### Lipid profile test

The blood samples were centrifuged at 3000 rpm and 2 °C for 15 minutes to separate blood plasma from serum. After centrifugation, the serum was collected in fresh, clean tubes and stored at −20 °C for the lipid profile assay, following the procedures outlined by Anyanwu et al. [30]. Commercial kits from Randox Laboratories Ltd., UK, were utilized to assay the concentrations of total cholesterol (TC), high-density lipoprotein (HDL), and total triglycerides (TG) in the serum. The concentration of low-density lipoprotein (LDL) was calculated using equation 1.


(1) LDL = TC–HDL– TG/2.2

### Liver function tests

The liver function tests were conducted following the detailed procedures outlined by Yahaya et al. [31]. The blood samples were allowed to clot at room temperature, following which the clots were removed by centrifuging the samples for 10 minutes at 2000 x g. The resulting supernatant (serum) was carefully poured into a pre-washed tube and used to determine the levels of liver enzymes, including alanine transaminase (ALT), aspartate transaminase (AST), alkaline phosphatase (ALP), albumin (ALB), and protein. ALT, AST, and ALP were estimated using ultraviolet, colorimetric, and spectrophotometric methods, respectively, while the biuret method was employed for the estimation of albumin (ALB) and total protein.

### Kidney function tests

The kidney function tests were conducted using blood serum, following the procedures outlined by Yahaya et al. [32]. Levels of serum sodium (Na^+^), potassium (K^+^), chloride (Cl^-^), calcium (Ca^2+^), urea, and creatinine were measured using an autoanalyzer (SKU: SM100).

### Data analysis

All analyses were performed using the Statistical Package for Social Science (SPSS) version 21 for Windows. The hematological parameters, lipid profile, as well as liver and kidney function parameters, were presented as mean ± standard deviation. The differences between the exposed and control groups were determined using Duncan test. Statistical significance was defined as p ≤ 0.05.

## Results

### Haematological parameters of the rats

Table 1 compares the hematological parameters of rats administered microplastic solutions with negative control rats (untreated rats) and positive control rats (rats treated with 10 % lead acetate). The results for white blood cells (WBC) show no significant difference (p < 0.05) among the treated rats and the control groups. However, the WBC counts of the treated rats decreased, with the highest concentration observed in the rats dosed with 1% (4.7 ± 0.28 x 10^9^L^-1^) and the lowest in the rats dosed with 10 % (4.2 ± 0.94 x 10^9^ L^-1^), in contrast to the negative control (4.7 ± 0.28 x 10^9^ L^-1^) and positive control (4.6 ± 0.66 x 10^9^L^-1^).

The lymphocytes (LYM) of all the treated rats decreased significantly (p < 0.05) compared to each other and the control groups. Rats fed with 10 % microplastics showed the lowest values (16.0 ± 1.32 %), while those dosed with 1 % showed the highest (20.6 ± 1.02 %), in comparison to the negative control (20.2 ± 0.89 %) and positive control (18.06 ± 0.94 %).

The monocytes to lymphocytes ratio (MLR) decreased, reaching as low as 8.6±0.21% in the group dosed with 10 %, with a significant difference (p < 0.05) compared to other groups, and peaked in the rats dosed with 5 % microplastics (11.7 ± 1.84 %), showing a significant difference (p < 0.05) compared to other groups except the negative control (11.6 ± 0.45 %).

Neutrophil (NEUT) concentrations reduced, reaching as low as 57.4 ± 0.44 % in the group dosed with 10 %, with a significant difference (p < 0.05) compared to other groups. Meanwhile, the group dosed with 1 % microplastics increased (69.1 ± 1.84 %) compared to the negative control (68.2 ± 1.39 %) and positive control (67.7 ± 1.63 %).

Red blood cells (RBC) showed no significant difference (p < 0.05) among all the groups. However, RBC counts decreased, reaching as low as 4.46 ± 1.80 x 10^12^ L^-1^ in the group dosed with 10 %, and peaked in the group dosed with 1 % (5.15 ± 0.84 x 10^12^ L^-1^), compared to the positive control (4.44 ± 0.46 x 10^12^ L^-1^) and negative control (5.12 ± 0.67 x 10^12^ L^-1^).

Hemoglobin (HGB) levels reduced, recording 11.3 g dL^-1^ in the rats administered 5 % and 11.2 g dL^-1^ in the rats dosed with 10 % microplastics with no significant difference (p < 0.05) between them. Rats administered 1 % had 11.8 ± 0.41 g dL^-1^ compared to 13.5 ± 2.12 g dL^-1^ of the positive control and 11.3 ± 0.46 g dL^-1^ of the negative control.

The hematocrits (HCT) results showed no significant difference (p < 0.05) between the treated groups and the positive control. However, HCT decreased, with 10 % being the lowest (37.6 ± 2.64 %) and 1 % being the highest (38.4 ± 1.13 %) compared to 43.7 ± 2.62 % and 35.8 ± 0.14 % of the negative and positive control, respectively.

The mean corpuscular volume (MCV) decreased, with the lowest values in the group administered 1 % (76.4 ± 0.34 fL) showing a significant difference (p < 0.05) compared to other groups, and the highest in the group dosed with 10 % (82.0 ± 1.63 fL) with no significant difference (p < 0.05) compared to the positive control (80.7 ± 3.26 fL).

The mean corpuscular hemoglobin (MCH) of all groups showed no significant difference (p < 0.05). However, MCH of the treated rats reduced, reaching as low as 22.9 ± 1.73 pg in the group dosed with 1 % and peaked in the group dosed with 10 % (25.8 ± 1.73 pg) against the negative control (26.3 ± 2.62 pg) and positive control (25.4 ± 1.33 pg).

The mean corpuscular hemoglobin concentration (MCHC) in all groups also showed no significant difference (p < 0.05). However, the MCHC of the rats dosed with 5 % and 10 % increased, having 31.1 ± 1.73 g dL^-1^ and 31.5 ± 1.08 g dL^-1^, respectively, while those dosed with 1 % decreased (30.7 ± 0.82 g dL^-1^) compared to 30.8 ± 1.13 and 31.5 ± 1.08 g dL^-1^ of the negative and positive control, respectively.

The platelet (PLT) results showed a significant difference (p < 0.05) among the groups. The PLT of the rats dosed with 1 % (262 ± 8.64 x 10^9^ L^-1^) and 5 % (284 ± 9.42 x 10^9^ L^-1^) increased, but those administered 10 % decreased (214 ± 9.93 x 10^9^ L^-1^) compared to the negative control (225 ± 10.8 x 10^9^ L^-1^) and positive control (168 ± 10.6 x 10^9^ L^-1^).

### Lipid profiles of the rats

**Figure 2.**
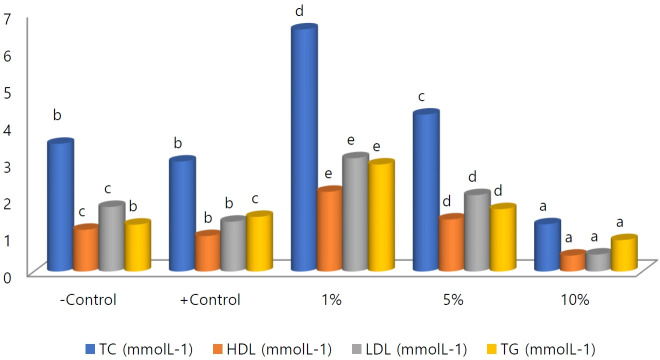
Fasting lipid profiles of the rats. Note: bars with different letters across the groups are significantly different according to Duncan test (p ≤ 0.05); - ve control = untreated rats; + ve control = rats fed 10 % lead acetate; 1 %, 5 %, and 10 % are rats fed with microplastics solutions; TC = total cholesterol; HDL = high-density lipoprotein; LDL = low-density lipoprotein; TG = triglyceride.

Figure 2 displays the lipid profiles of the treated rats in comparison to negative control and positive control rats. Significant differences (p < 0.05) were observed among the treated groups and control groups in all lipid profile parameters. The total cholesterol (TC) of rats treated with 1 % and 5 % increased, reaching 6.55 ± 0.28 and 4.25 ± 0.47 mmol L^-1^, respectively, but decreased in those dosed at 10 % (1.28 ± 0.03 mmol L^-1^) compared to 3.46 ± 0.78 mmol L^-1^ of the negative control and 2.98 ± 0.04 mmol L^-1^ of the positive control.

Similarly, high-density lipoprotein (HDL) increased in the groups administered 1 % (2.16 ± 0.06 mmol L^-1^) and 5 % (1.41 ± 0.04 mmol L^-1^) but decreased in the group dosed at 10 % (0.43 ± 0.05 mmol L^-1^) compared to the negative control (1.13 ± 0.18 mmol L^-1^) and positive control (0.96 ± 0.03 mmol L^-1^).

Low-density lipoprotein (LDL) also increased in the groups administered 1 % and 5 %, with values of 3.07 ± 0.06 mmol L^-1^ and 2.07 ± 0.04 mmol L^-1^ respectively, and decreased in the 10 % group (0.46 ± 0.04 mmol L^-1^) compared to 1.75 ± 0.04 mmol L^-1^ and 1.35 ± 0.04 mmol L^-1^ of the negative and positive control, respectively.

Total triglycerides (TG) increased in the groups dosed at 1 % and 5 %, recording 2.90 ± 0.06 mmol L^-1^ and 1.69 ± 0.05 mmol L^-1^, respectively, but reduced in the group administered with 10 % (0.85 ± 0.01 mmol L^-1^) compared to 1.27 ± 0.08 mmol L^-1^ and 1.48 ± 0.01 mmol L^-1^ of the negative and positive control, respectively.

### Effects of microplastic on liver functions

Table 2 presents a comparison of liver function parameters in treated rats with negative control and positive control rats. Significant difference (p < 0.05) were observed among the treated groups and control groups in all the liver functions parameters The total protein (TP) of the treated rats increased, with rats dosed at 5 % exhibiting the highest values (69.00 ± 2.50 gL^-1^) and those administered 10 % showing the least (65.00 ± 0.00 gL^-1^), in contrast to 64.00 ± 2.00 gL^-1^ and 68.00 ± 1.00 gL^-1^ of the negative and positive control, respectively.

Albumin (ALB) increased, with rats fed 5 % having the highest values (37.00 ± 1.30 gL^-1^) and 10 % rats having the least (32.00 ± 2.00 gL^-1^), compared to the negative control (29.00 ± 1.40 gL^-1^) and positive control (36.00 ± 2.50 gL^-1^).

Aspartate transaminase (AST) increased, with rats dosed at 1 % (38.00 ± 1.00 U L^-1^) having the highest value, and rats dosed at 5 % having the least (32.00 ± 1.00 U L^-1^), compared to the negative control (10.00 ± 1.00 U L^-1^) and positive control (46.00 ± 2.00 U L^-1^).

Alanine transaminase (ALT) increased, with rats dosed at 1 % recording the highest values (33.00 ± 1.72 U L^-1^), and rats fed 10 % having the least (27.00 ± 0.55 U L^-1^), compared to 8.00 ± 1.00 U L^-1^ and 40.00 ± 2.41 U L^-1^ of the negative and positive control, respectively.

Alkaline phosphatase (ALP) of rats fed 10 % increased (37.00 ± 0.00 U L^-1^), but rats fed 1 % and 5 % decreased, both having 32.00 ± 1.20 U L^-1^ and 30.00 ± 1.10 U L^-1^, respectively, compared to the negative control (34.00 ± 1.30 U L^-1^) and positive control (40.00 ± 1.10 U L^-1^).

### Effects of microplastics on renal functions

Table 3 presents a comparison of kidney function parameters in treated rats with negative and positive control rats. The sodium ion (Na^+^) of the treated rats increased, with rats administered 1 % having the highest values (144 ± 1.40 mmol L^-1^) and rats dosed at 5 % having the least (142 ± 2.00 mmol L^-1^) with significant difference (p < 0.05) between them, in contrast to 136 ± 1.00 mmol L^-1^ and 141 ± 2.00 mmol L^-1^ of the negative and positive control, respectively.

Compared with negative control, no significant difference (p < 0.05) was observed among the values of potassium ion (K^+^) of all groups. But K^+^ increased across all groups, with rats dosed at 10 % having the highest (6.70 ± 0.52 mmol L^-1^) and rats dosed at 1 % recording the least (6.20 ± 1.45 mmol L^-1^), compared to the negative control (3.90 ± 1.20 mmol L^-1^) and positive control (6.50 ± 0.55 mmol L^-1^).

Also, no significant difference (p < 0.05) was observed among the values of chloride ion (Cl^-^) of all groups. However, Cl^-^increased across all groups, with rats dosed at 1 % and 10 % jointly having the highest values of 106 mmol L^-1^ each, and the 5 % group recording the least (105 ± 0.47 mmol L^-1^), compared to 98 ± 4.22 mmol L^-1^ and 104 ± 0.33 mmol L^-1^ of the negative and positive control, respectively.

Urea of the rats dosed at 1 % and 5 % increased, with values of 6.10 ± 1.17 mmol L^-1^ and 6.30 ± 0.37 mmol L^-1^, respectively, but rats dosed at 10 % decreased (4.0 ± 7.87 mmol L^-1^), compared to 5.70 ± 2.07 mmol L^-1^ and 5.60 ± 0.23 mmol L^-1^ of the negative and positive control, respectively with significant difference (p < 0.05) among the groups.

In addition, significant difference (p < 0.05) was observed among the values of creatinine (CRT) of all groups. CRT of the treated rats was elevated across all groups, with rats fed 10 % having the highest (91 ± 1.55 μmol L^-1^) and those fed 5 % recording the least (64 ± 1.88 μmol L^-1^), compared to negative control (53 ± 4.55 μmol L^-1^) and positive control (102 ± 2.00 μmol L^-1^).

## Discussion

The current study aimed to determine the effects of microplastics exposure by evaluating several health indices in exposed albino rats (*Rattus norvegicus*). These indices included hematological parameters, lipid profiles, as well as liver and kidney function parameters. The research became necessary due to increasing concerns over the widespread distribution of microplastics, their potential toxicities, and the lack of studies on the nature of their effects. Lead acetate was used as positive control in this study to establish that the effects observed in the rats exposed to microplastics are caused by a toxicant. Lead acetate exposure has been demonstrated by Ilesanmi et al. [28] to cause hematological and biochemical damage.

Table 1 reveals alterations in the hematological parameters of the treated rats compared with the negative control (untreated rats). Similar alterations were observed in the hematological parameters of rats treated with 10 % lead acetate (positive control rats), a known toxicant as mentioned earlier, confirming that the changes in hematological parameters induced by microplastics must be attributed to toxicities. Specifically, the white blood cells (WBC) and its subtypes, lymphocytes and neutrophils, in treated rats decreased at higher microplastic concentrations (5 % and 10 %), indicating immunosuppressive effects of microplastics, as revealed by Rajendran and Chandrasekaran [35]. According to the authors, microplastics exposure can cause cellular cytotoxicity, oxidative stress, and reduction in WBC. Moreover, Buonacera et al. [36] postulate that neutrophils, in particular, are responsible for the first line of the host immune response against invading antigens, and phagocytosis of microplastics elicits an inflammatory response [35], which may result in the destruction of neutrophils and monocytes. Inflammation leading to the destruction of monocytes is evident in the elevated monocyte-to-lymphocytes ratio (MLR) of the treated rats. According to Zheng et al. [37], an elevated MLR is an indication of systemic inflammation, resulting in the decline of monocytes. These findings are consistent with those obtained by Hamed et al. [38] and Ammar et al. [39], who observed a decline in WBC counts of animals following microplastic exposure. Contrastingly, Hasan et al. [40] reported elevated WBC in Nile tilapia exposed to polyamide microplastics. These inconsistencies could be attributed to various factors. According to Li et al. [41] and Shen et al. [42], the toxic effects of microplastics are complex and are affected by many factors, including physical and chemical properties, exposure time, additives, size, shape, surface charge, weathering/aging process, and adsorption, etc. Differences in susceptibility to environmental stress among species, even within the same species, may also influence the effects of microplastic exposure.

Table 1 further shows that the red blood cells (RBC), hemoglobin (HGB), hematocrit (HCT), and platelet (PLT) levels of the treated rats decreased, similar to the rats treated with lead acetate. This reduction indicates anemia, possibly modulated by oxidative stress as well as genotoxic and cytotoxic effects induced by microplastics [35]. Previous studies by Abdel-Zaher et al. [43] and Choi and Kim [44] also noticed a reduction in the hematological parameters of mice and fish exposed to polyethylene and polyamide microplastics, respectively. Additionally, Hamed et al. [38] reported a decline in the hematological indices (RBC, HGB, HCT, MCHC, and PLT) of Nile tilapia exposed to microplastics. However, unlike the current study, Hamed et al [38] observed a raised mean corpuscular volume (MCV) and mean corpuscular hemoglobin (MCH) in the exposed fish. A reduced MCV observed in the treated rats in the current study indicates that their RBC size was smaller than normal and may indicate microcytic anemia due to inflammation [45,46]. Additionally, reduced MCH was noted among the treated rats, which may indicate hypochromic anemia. Moreover, the decreased mean corpuscular hemoglobin concentrations (MCHC) observed among the treated rats may be associated with hypochromic anemia connected to cancer [47].

Compared with the non-treated rats, microplastics caused alterations in the lipid profile of the treated rats (Table 2). The parameters evaluated include total cholesterol (TC), triglycerides (TG), high-density lipoprotein (HDL), and lowdensity lipoprotein (LDL). However, while the rats dosed with 1% or 5% microplastic solution exhibited an increased lipid profile, the rats treated with 10% significantly revealed a decreased lipid profile similar to what was observed in the rats treated with 10% lead acetate. The increase in the lipid profile is termed hyperlipidemia, possibly caused by a dietary disorder [48] or renal failure or physical inactivity [49] induced by microplastics. The decrease observed in the lipid profile of rats dosed with 10% microplastic solution is termed hypolipidemia, possibly caused by liver dysfunction or chronic inflammation or malabsorption or malnutrition [50, 51] induced by microplastics ingestion. Feeding habits, in particular, have been demonstrated by Cole et al. [52] to alter in Coldwater copepod following microplastic exposure, resulting in an altered lipid profile. The results obtained are consistent with those of Deng et al. [53] and Liu et al. [54], both of whom reported an increase in the lipid profile of mouse livers following microplastic exposures. Similarly, Nnoruka et al. [55] reported a decrease in HDL and an increase in LDL in rats following microplastic exposure. Banaee et al. [56] observed an increase in TC of turtles exposed to microplastics. Contrastingly, Smith et al. [57] did not observe any significant change in the lipid profiles of quill birds exposed to microplastics. The possible reasons for these inconsistencies had been suggested earlier.

The evaluation of the liver enzymes revealed that the tested parameters, including total protein (TP), albumin (ALB), aspartate transaminase (AST), alanine transaminase (ALT), and alkaline phosphatase (ALP) showed a significant increase across the groups compared with the untreated rats, except for the ALP of the rats dosed with 1 % and 5 % microplastic solutions. The rats dosed with 10 % lead acetate also exhibited the same trend as the treated rats, confirming that the increase probably emanated from toxicities. However, their values remained within normal range, except for AST and ALT. The increase in the liver enzymes of the rats is symptomatic of the presence of cytotoxic agents, which were particularly more pronounced in the rats dosed with 10% microplastic solutions. According to Yahaya et al. [31], the membrane of hepatocytes (liver cells) is highly permeable, so when the liver is injured, the liver enzymes are released into the bloodstream, raising the levels of the enzymes in the blood. The liver is the primary target of toxins because it processes all substances ingested. The findings of the current study align with those of Yang et al. [21], who reported altered liver enzymes in red tilapia exposed to microplastics. Hamed et al. [38] also observed a significant increment in the biochemical parameters of Nile tilapia (AST, ALT, ALP, glucose, total protein, albumin, and globulin) after exposure to microplastics for 15 days compared to the control group in a dose-dependent manner. However, in a study on microplastic exposure in tilapia fish by Raza et al. [58], TP contents were expressively increased, while other blood parameters (AST, ALP, ALT) were significantly decreased.

Although the kidney function parameters of the treated rats show a significant elevation in sodium ion (Na^+^), potassium ion (K^+^), chloride ion (Cl^-^), urea, and creatinine (CRT) compared with the control rats, their values remained within normal range limits, except for K^+^ (Table 4). The rats administered with 10 % lead acetate also showed significantly elevated kidney function parameters, indicating that the alterations observed in the rats treated with the microplastic solution could be induced by toxicities. The changes observed in the kidney function parameters are indicative of kidney toxicity or injury. This finding is consistent with Ahmed et al. [59], who found a significant increase and alteration in kidney parameters when compared with the control groups. Banaee et al. [56] reported an increase in creatinine, urea, and calcium (Ca+2) in turtles exposed to microplastics compared with the control group. Hamed et al. [38] also reported raised glucose, cholesterol, creatinine, and uric acid levels in Nile tilapia exposed to microplastics. However, in a study by Shen et al. [42], microplastics administration decreased the levels of blood urea nitrogen in mice, while serum creatinine and uric acid concentrations were unaffected. The possible reasons for these discrepancies have been explained earlier.

It is noteworthy that the characteristics of the experimental microplastics were similar to those of microplastics detected in the environment of the study area, as revealed in a study on microplastics abundance and characteristics in borehole water in the city by Yahaya et al. [60]. This suggests that the effects observed in the experimental animals are likely to occur in animals exposed to microplastics in the environment. However, the study was conducted in the animal house, where normal weather conditions were maintained, compared to the extremely hot and harsh conditions of the study area environment, as described in the study site section. This indicates that the effects of microplastics on animals exposed to them in the study area environment might be worse than those observed in the experimental animals.

## Conclusions

The results revealed that microplastics altered the hematological parameters of the exposed rats, with those administered the highest concentration (10 %) being the most affected. Specifically, it decreased the levels of hematological parameters, which are signs of anemia. Furthermore, at low-to-medium concentrations ( 1 % and 5 %), microplastics raised the lipid profile levels of the treated rats, while at a high concentration (10 %), it decreased them, causing hyperlipidemia and hypolipidemia, respectively. Microplastics also increased the levels of liver and kidney function parameters, suggesting liver and kidney injuries. Overall, the results show that microplastics can cause adverse health effects on exposed animals, including humans, particularly affecting hematobiochemical parameters. These findings suggest that efforts should be made to minimize microplastics exposure. We recommend more studies to ascertain our claims, particularly on histopathological assessments, since the liver and kidney parameters suggest histopathological damage.

## Declaration of generative AI and AI-assisted technologies in the writing process

During the preparation of this work the authors used ChatGPT in order to correct grammatical errors and make sentences flow. After using this tool, the authors reviewed and edited the content as needed and take full responsibility for the content of the publication.

## Figures and Tables

**Table 1.** Haematological parameters of the rats.

Parameters	- Control	+ Control	1 %	5 %	10 %
WBC (x10^9^ L^-1^)	4.7 ± 0.28^a^	4.6 ± 0.66^a^	4.7 ± 0.28^a^	4.4 ± 0.85^a^	4.2 ± 0.94^a^
LYM (%)	20.2 ± 0.89^b^^c^	18.6 ± 0.94^b^^c^	20.6 ± 1.02^c^	18.2 ± 1.35^b^	16.0 ± 1.32^a^
MLR (%)	11.6 ± 0.45^b^	9.7 ± 0.21^a^^b^	10.3 ± 1.63^a^^b^	11.7 ± 1.84^b^	8.6 ± 0.21^a^
NEUT (%)	68.2 ± 1.39^b^	67.7 ± 1.63^b^	69.1 ± 1.84^b^	59.0 ± 1.63^a^	57.4 ± 0.44^a^
RBC (x10^12^ L^-1^)	5.12 ± 0.67^a^	4.44 ± 0.46^a^	5.15 ± 0.84^a^	5.83 ± 1.93^a^	5.81 ± 1.80^a^
HGB (gd L^-1^)	13.5 ± 2.12^a^^b^	11.3 ± 0.46^a^	11.8 ± 0.41^a^^b^	15.0 ± 2.16^b^	15.0 ± 2.16^b^
HCT (%)	43.7 ± 2.62^b^	35.8 ± 0.14^a^	38.4 ± 1.13^a^	38.1 ± 2.21^a^	37.6 ± 2.64^a^
MCV (fL)	85.5 ± 4.71^b^	80.7 ± 3.26^a^^b^	76.4 ± 0.34^a^	82.6 ± 2.30^b^	82.0 ± 1.63^b^
MCH (pg)	26.3 ± 2.62^a^	25.4 ± 1.33^a^	22.9 ± 1.73^a^	25.7 ± 2.09^a^	25.8 ± 1.73^a^
MCHC (gd L^-1^)	30.8 ± 1.13^a^	31.5 ± 1.08^a^	30.7 ± 0.82^a^	31.1 ± 1.73^a^	31.5 ± 1.08^a^
PLT (x10^9^ L^-1^)	225 ± 10.8^b^	168 ± 10.6^a^	262 ± 8.64^c^	284 ± 9.41^d^	214 ± 9.93^b^

Note: values were expressed as mean± standard deviation (n = 5); - ve control = untreated rats; + ve control = rats fed 10 % lead acetate; 1 %, 5 %, and 10 % are rats fed with microplastics solutions; means followed by different letters within each row are significantly different according to Duncan test (p ≤ 0.05) and vice versa; WBC = white blood cells; LYM = lymphocytes; MLR = monocytes-to-lymphocytes ratio; NEUT = neutrophils; RBC = red blood cells; HGB = haemoglobin; HCT = haematocrit; MCV = mean corpuscular volume; MCH = mean corpuscular haemoglobin; MCHC = mean corpuscular haemoglobin concentration; PLT = platelets..

**Table 2.** Liver function parameters of the rats.

Groups	TP (gL^-1^)	ALB (gL^-1^)	AST (U L^-1^)	ALT (U L^-1^)	ALP (U L^-1^)
- ve	64.00 ± 2.00^a^	29.00 ± 1.40^a^	10.00 ± 1.00 ^a^	8.00 ± 1.00^a^	34.00 ± 1.30^c^
+ ve	68.00 ± 1.00^b^^c^	36.00 ± 2.50^c^^d^	46.00 ± 2.00^d^	40.00 ± 2.41^d^	40.00 ± 1.10^e^
1 %	66.00 ± 1.00^a^^b^	33.00 ± 1.55^b^^c^	38.00 ± 1.00^c^	33.00 ± 1.72^c^	32.00 ± 1.20^b^
5 %	69.00 ± 2.50^c^	37.00 ± 1.30 ^d^	32.00 ± 1.00 ^b^	27.00 ± 1.20^b^	30.00 ± 1.10^a^
10 %	65.00 ± 0.00 ^a^	32.00 ± 2.00^a^^b^	33.00 ± 0.00^b^	27.00 ± 0.55^b^	37.00 ± 0.00^d^
Normal range [33]	60 - 80 gL^-1^	30 - 50gL^-1^	12 U L^-1^	12 U L^-1^	40 - 150 U L^-1^

Note: values were expressed as mean ± standard deviation (n = 5); means followed by different letters within each row are significantly different according to Duncan test (p ≤ 0.05) and vice versa; - ve control = untreated rats; + ve control = rats fed 10 % lead acetate; 1 %, 5 %, and 10 % are rats fed with microplastics solution; ALP = alkaline phosphatase; AST = aspartate transaminase; ALT = alanine transaminase; TP = Total protein; ALB = albumin.

**Table 3.** Kidney renal function parameters of albino rats.

Parameter	Na+ mmolL^-1^	K+ mmolL^-1^	Cl- mmolL^-1^	Urea mmolL^-1^	CRT µmolL^-1^
- ve	136 ± 1.00^a^	3.90 ± 1.20^a^	98 ± 4.22^a^	5.70 ± 2.07^a^^b^	53 ± 4.55^a^
+ ve	141 ± 2.00^b^	6.50 ± 0.55^b^	104 ± 0.33^a^	5.60 ± 0.23^a^^b^	102 ± 2.00^e^
1 %	144 ± 1.40^c^	6.20 ± 1.45^b^	106 ± 1.41^a^	6.10 ± 1.17^a^^b^	82 ± 6.22^c^
5 %	142 ± 2.00^b^^c^	6.60 ± 0.50^b^	105 ± 0.47^a^	6.30 ± 0.37^b^	64 ± 1.88^b^
10 %	143 ± 0.00^b^^c^	6.70 ± 0.52^b^	106 ± 8.12^a^	4.0 ± 0.87^a^	91 ± 1.55^d^
Normal [34]	135 - 145	3.5 - 5.5	95 - 110	2.5 - 8.5	10 – 90

Note: values were expressed as mean ± standard deviation (n = 5); means followed by different letters within each row are significantly different according to Duncan test (P ≤ 0.05) and vice versa; - ve control = untreated rats; + ve control = rats fed 10 % lead acetate; 1 %, 5 %, and 10 % are rats fed with microplastics solution values; Na+= sodium ion; K+ = potassium ion; Cl- = chloride ion; CRT = creatinine.
